# Removal of Integrated Hepatitis B Virus DNA Using CRISPR-Cas9

**DOI:** 10.3389/fcimb.2017.00091

**Published:** 2017-03-22

**Authors:** Hao Li, Chunyu Sheng, Shan Wang, Lang Yang, Yuan Liang, Yong Huang, Hongbo Liu, Peng Li, Chaojie Yang, Xiaoxia Yang, Leili Jia, Jing Xie, Ligui Wang, Rongzhang Hao, Xinying Du, Dongping Xu, Jianjun Zhou, Mingzhen Li, Yansong Sun, Yigang Tong, Qiao Li, Shaofu Qiu, Hongbin Song

**Affiliations:** ^1^Center for Infectious Disease Control, Institute of Disease Control and Prevention, Academy of Military Medical SciencesBeijing, China; ^2^State Key Laboratory of Pathogen and Biosecurity, Beijing Institute of Microbiology and EpidemiologyBeijing, China; ^3^Research Centre for Liver Failure, Beijing 302nd HospitalBeijing, China; ^4^Research Center for Translational Medicine, Cancer Stem Cell Institute, East Hospital, Tongji University School of MedicineShanghai, China; ^5^Gladcan Consulting CompanyBeijing, China; ^6^Research and Development Department, Beijing Center for Physical and Chemical AnalysisBeijing, China; ^7^Department of Surgery, University of MichiganAnn Arbor, MI, USA

**Keywords:** hepatitis B virus, CRISPR-Cas9, integrated HBV DNA, HBV cccDNA, whole genome sequencing

## Abstract

The presence of hepatitis B virus (HBV) covalently closed circular DNA (cccDNA) and the permanent integration of HBV DNA into the host genome confers the risk of viral reactivation and hepatocellular carcinoma. Nucleoside/nucleotide analogs alone have little or no capacity to eliminate replicative HBV templates consisting of cccDNA or integrated HBV DNA. Recently, CRISPR/Cas9 technology has been widely applied as a promising genome-editing tool, and HBV-specific CRISPR-Cas9 systems were shown to effectively mediate HBV cccDNA disruption. However, the integrated HBV DNA fragments are considered as important pro-oncogenic properties and it serves as an important template for viral replication and expression in stable HBV cell line. In this study, we completely excised a full-length 3,175-bp integrated HBV DNA fragment and disrupted HBV cccDNA in a stable HBV cell line. In HBV-excised cell line, the HBV cccDNA inside cells, supernatant HBV DNA, HBsAg, and HBeAg remained below the negative critical values for more than 10 months. Besides, by whole genome sequencing, we analyzed off-target effects and excluded cell contamination. It is the first time that the HBV infection has been fully eradicated in a stable HBV cell line. These findings demonstrate that the CRISPR-Cas9 system is a potentially powerful tool capable of promoting a radical or “sterile” HBV cure.

## Introduction

Chronic hepatitis B (CHB) is a major public health problem worldwide, with 350–400 million chronic HBV (hepatitis B virus) carriers (Seo and Yano, 2014). HBV infection results in 0.5–1 million deaths per year due to cirrhosis of the liver, hepatocellular carcinoma, and liver failure (Kao and Chen, 2002; Roberts and Gores, 2005). HBV covalently closed circular DNA (cccDNA) is very stable in CHB patients and serves as the template for viral mRNA and pre-genomic RNA synthesis (Moraleda et al., 1997; Dandri et al., 2000; Gish et al., 2015; Guo and Guo, 2015; Nassal, 2015). The integration of HBV DNA into the host genome can cause alterations of the host genome, leading to changes in genes associated with cell proliferation, differentiation, and survival (Bonilla Guerrero and Roberts, 2005; Feitelson and Lee, 2007; Jiang S. et al., 2012; Jiang Z. et al., 2012; Sung et al., 2012; Xu et al., 2014). HBV DNA integration is also an important factor in hepatocarcinogenesis (Bréchot, 2004; Sung et al., 2012; Tarocchi et al., 2014; Xu et al., 2014). Current anti-HBV treatments with either nucleoside/nucleotide analogs (NAs) or interferon do not cure CHB, and relapses are common (Bang and Kim, 2014). Although NAs can inhibit viral reverse transcriptase and suppress HBV replication, these drugs alone have little or no ability to eliminate replicative HBV templates comprising cccDNA (Moraleda et al., 1997; Dandri et al., 2000) or integrated HBV DNA (Zucman-Rossi and Laurent-Puig, 2007). Given the shortcomings of current therapeutic options, there is a need to approach CHB in a fundamentally different way (Shaw et al., 2006; Sharon and Chu, 2008).

A new genome-editing tool, CRISPR-Cas9, was recently developed based on the bacterial immune system's clustered regularly interspaced short palindromic repeats (CRISPRs) (Qi et al., 2013). Zinc Finger Nucleases (ZFNs) and Transcription activator-like effectors nucleases (TALENs) have been under investigation for their capacity to specifically disrupt HBV genomes *in vitro* and *in vivo* (Cradick et al., 2010; Bloom et al., 2013; Chen et al., 2014). However, compared with ZFNs and TALENs, the CRISPR/Cas9 system can be more easily reprogrammed and delivered both *in vitro* and *in vivo* to cleave virtually any DNA sequence by simply redesigning the guide RNAs (gRNAs), which is predicated to be a promising genome-editing tool with broad applications (Qi et al., 2013; Ran et al., 2013; Zhang et al., 2014). Using CRISPR-Cas9, Hu et al. completely excised the full length of integrated HIV proviral DNA in a stable HIV monoclonal cell line (Hu et al., 2014). In these studies, HBV-specific CRISPR-Cas9 systems effectively mediated gene disruption in HBV templates in expression vectors (Lin et al., 2014; Liu et al., 2015) and HBV cccDNA (Seeger and Sohn, 2014; Kennedy et al., 2015; Zhen et al., 2015a) both *in vitro* and *in vivo*. However, none of these studies demonstrated removal of the full-length integrated HBV DNA and subgenomic integrated HBV DNA fragments in a stable HBV cell line (Lin et al., 2014; Seeger and Sohn, 2014; Kennedy et al., 2015; Liu et al., 2015; Zhen et al., 2015a). These shortcomings largely limited the prospect of developing a fundamental therapeutic method of viral eradication through CRISPR-Cas9. Several studies have proposed that removal of integrated HBV DNA from the host genome is a necessary measure to recover the stability of the chromosome and cure HBV-related HCC (Peng et al., 2015; Ramanan et al., 2015; Wang et al., 2015). In a pioneering study, Karimova et al. disrupted integrated HBV DNA, using an integrated HBV reporter sequence in HeLa and HEK293 cell lines (Karimova et al., 2015). In HBV-infected cells, the existence of many different forms of episomal HBV DNA (Tuttleman et al., 1986) and multiple integrated HBV sites in different chromosomes (Matsubara and Tokino, 1990) made it cumbersome to specifically amplify the full length of integrated HBV DNA sites in a stable HBV cell line, using Alu-/LM-PCR. Also, the limitations of the short reads generated by next-generation sequencing (NGS) meant that HBV DNA integration sites could only be inferred from paired-end reads containing both human and viral sequences (Hai et al., 2014). In a previous study, we established a stable HBV cell line, HepG2.A64 (CCTCC C 201163), using genotype C HBV strains (GenBank: HQ638218.1) isolated from hepatitis B patients (Wei-ming et al., 2014). Compared with HepG2.2.15, this cell line could produce more antigens, virions, and HBV cccDNA, and was easier to cultivate and transfect. HBV transfected in HepG2.A64 cells contained entecavir-resistant mutations, which had already been used in drug resistance investigations (Liu et al., 2016). Moreover, we employed CRISPR-Cas9 to disrupt HBV cccDNA and inhibit viral replication in this cell line in our previous study (Li et al., 2016). In this study, not only HBV cccDNA, we also removed the full length of integrated HBV DNA, which means that we achieved a “sterile” eradication of HBV infection in this stable HBV cell line.

## Materials and methods

### Plasmid preparation

The Cas9/gRNA dual-expression vector pSpCas9(BB)-2A-Puro (PX459) was a gift from Feng Zhang (Addgene plasmid #48139) and was constructed according to a previously described protocol (Ran et al., 2013). Using a gRNA prediction tool (http://crispr.mit.edu/), five candidate target sequences were derived from the HBV genome (GenBank accession number HQ638218). These protospacers were subsequently inserted into dual-expression vectors under the control of the U6 promoter. Plasmids were purified using the EndoFree Plasmid Maxi Kit (Qiagen, Germany).

### Cell culture and transfections

All cells were maintained in Dulbecco's modified Eagle medium supplemented with 10% fetal calf serum at 37°C and 5% CO_2_. The HepG2.A64 (CCTCC C 201163) monoclonal cell line was established from HepG2 cells transfected with an HBV plasmid pTriexHBV1.1 containing 1.1 copies of HBV DNA (GenBank: HQ638218.1) (Wei-ming et al., 2014). This monoclonal cell line sustainably produced HBsAg, HBeAg, and HBV DNA. A total of 2 × 10^6^ A64 cells were seeded onto 10-cm plates 24 h before transfection. The Cas9/gRNA co-expression vectors were transfected into A64 cells using Lipofectamine LTX (Life Technologies, US). Puromycin (1 μg/μl) was used to increase transfection efficiency (Figure S1). At 72 h after transfection, transfected cells were seeded onto 24-well-plates (~4 × 10^4^ cells per well) for subsequent analysis. For subcloning, stable clones were subcultured after transfection and underwent puromycin selection by a limiting dilution method in 96-well-plates, and single cell-derived subclones were maintained for further studies.

### T7EI assay

The genomic region surrounding the target regions A and B was amplified by PCR; specific primers were designed with primer premier 5.0 (Table S1). All PCR products were verified by Sanger sequencing and subjected to a re-annealing process to induce heteroduplex formation. After re-annealing, products were treated with T7EI (New England Biolabs, US) at 37°C for 30 min and analyzed using 3% agarose gels. Gels were imaged using a gel imaging system (Bio-Rad, US).

### Immunoassay and qPCR

HBsAg and HBeAg were measured in the cell supernatants, using an ARCHITECT i2000 SR system (Abbott, US); data were reported in IU/ml for HBsAg and S/CO for HBeAg. Cell culture supernatants were extracted and tested for the presence of HBV DNA by real-time PCR. To quantitate HBV cccDNA, circular duplex DNA was subjected to PlasmidSafe ATP-dependent DNase (Epicentre, US), which has low activity toward HBV rcDNA, dsDNA, and ssDNA. Rolling circle amplification (RCA) was then performed to selectively amplify circular DNA. Based on the RCA products, HBV cccDNA was amplified and quantitated by TaqMan real-time PCR using specific cccDNA-selective primers and a probe targeting the gap region between DR1 and DR2 of the HBV genome (Zhong et al., 2011). To detect genomic DNA contamination, primer sets that amplify the HBV S DNA or control cellular genomic sequences located in the A1AT gene were used (Table S1). To quantitate the cell number, specific primers for human β-actin were used for real-time PCR.

### Whole-genome sequencing and bioinformatics analysis

Genomic DNA was isolated by a cetyltrimethylammonium bromide (CTAB)-based extraction method. DNA samples and genomic DNA libraries were prepared by the NGS facility at the Biomarker Technologies Company. All libraries were sequenced with paired-end 150-bp reads in two Illumina Rapid Run flow cells, using a HiSeq X 10 instrument (Illumina). Demultiplexed read data from the sequenced libraries were sent to the Biomarker Technologies Company for bioinformatics analysis. Briefly, the raw reads were mapped against the human genome (hg19), using BWA. A genomic analysis toolkit (GATK, version 2.8.1) was used for removal of duplicate reads, local alignment, base quality recalibration, and indel calling.

### Statistical analysis

For statistical analysis, Student's *t*-test was performed using the SAS software suite. *P* < 0.05 was considered significant. Error bars represent the SEM of at least three independent experiments.

## Results

### Analysis of integrated HBV DNA and rationale for selection of the CRISPR-Cas9 target site

**Figure 1:**
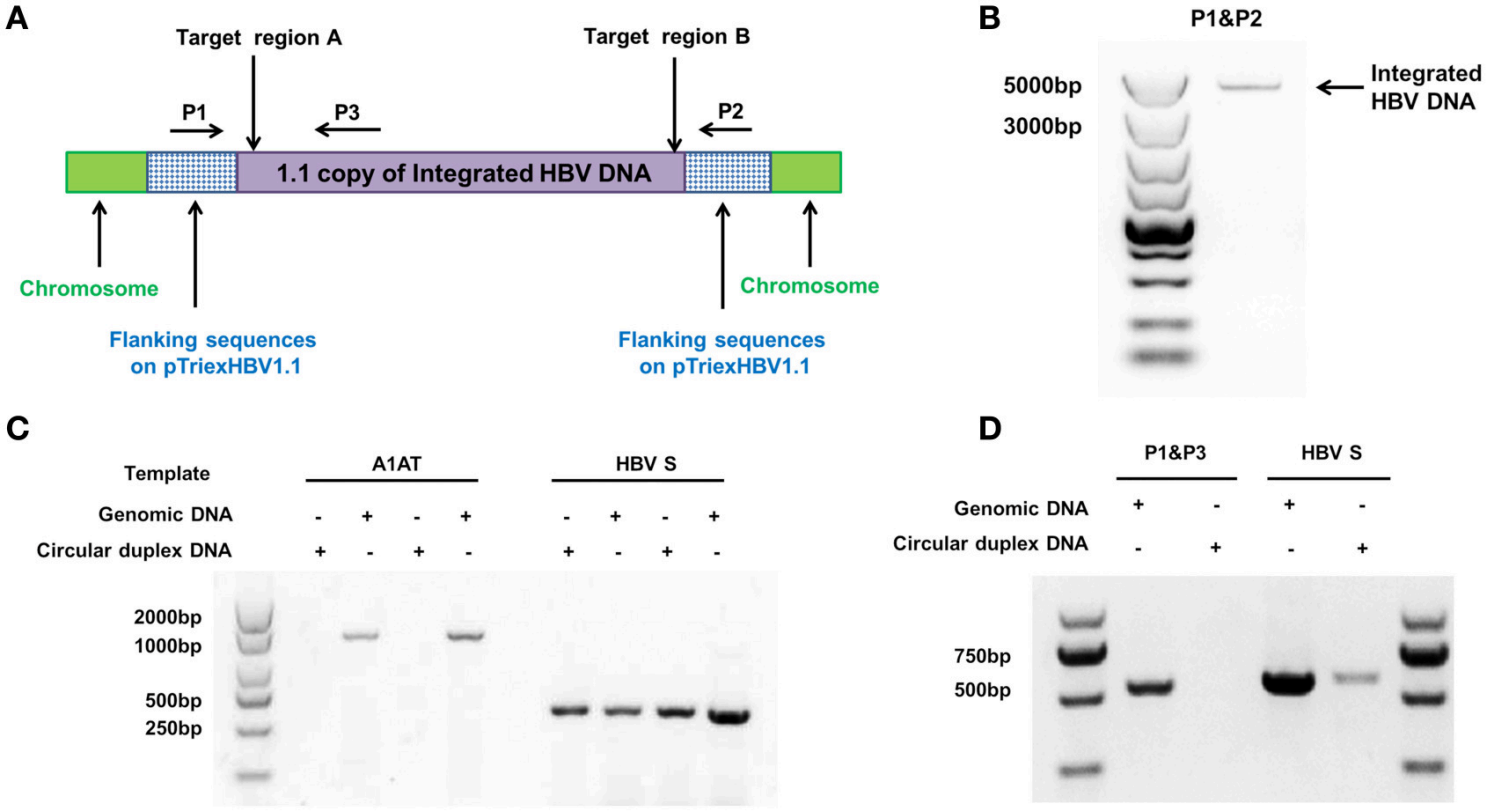
**Analysis of integrated HBV DNA in the stable HBV cell line A64. (A)** Integrated HBV DNA in the stable HBV A64 cell line and the gRNA target sites in the repeat region of the 1.1 HBV genome copy. **(B)** The 4,049-bp DNA fragment representing the 3,362-bp integrated HBV DNA (1.1 copies) plus a 687-bp pTriexHBV1.1-derived flanking sequence was efficiently amplified from cellular genomic DNA using the integrated HBV-specific primers P1 and P2. **(C)** PCR analysis using the A1AT and HBV S-gene primer sets conducted on total genomic DNA and circular duplex DNA to assess the effect of extraction on circular duplex DNA. **(D)** PCR analysis of the integrated HBV DNA or circular duplex DNA isolated from cells using the P1/P3 and S-gene primer sets. Using P1 and the HBV core region-specific primer P3, the HBV S-gene amplicons were predicted to be 542- and 572-bp, respectively. Primers P1/P3 did not amplify circular duplex DNA.

We used the stable HBV cell line HepG2.A64 (CCTCC C 201163, hereafter referred to as “A64”) as a cell model. The full length of integrated HBV DNA in this cell line was dependent on a foreign promoter (CMV chicken β-actin promoter) instead of viral promoters, which enabled amplification of the full-length replication-competent integrated HBV DNA, using a specific primer (P1) located in the foreign promoter region (Figure 1A). To ensure that the PCR products of the primers (P1 and P2) were the integrated HBV DNA rather than the fragment on pTriexHBV1.1, we used Plasmid-Safe ATP-Dependent DNase (PSAD) to extract the circular duplex DNA. HBV-specific primers (HBSF&R) and genome-specific primers (A1ATF&R) were used as the positive and negative controls, respectively, to evaluate the effect of the circular duplex DNA on extraction. The P1 primer and the HBV S gene-specific primer P3 did not amplify the circular duplex DNA (Figures 1C,D), indicating that there was no circular pTriexHBV1.1 in the stable HBV cell line A64 and that primer P1 was an integrated HBV DNA-specific primer. Next, we performed long-range PCR, using A64 genomic DNA with integrated HBV DNA-specific primers (P1 and P2) with a Phusion High-Fidelity PCR Kit (NEB, US), following the manufacturer's protocol. Sequencing of the PCR products revealed a 4,049-bp DNA fragment representing the 3,362-bp integrated HBV DNA (1.1 copies) plus a flanking 687-bp pTriexHBV1.1-derived sequence (Figure 1B). The 3,362-bp integrated HBV DNA contained an entire 3,173-bp HBV genome and a 189-bp repeat sequence of the HBV core region. To remove the full-length integrated HBV DNA, we employed one gRNA targeting the two repeat regions of the integrated HBV DNA, which was expected to be more efficient in transfection and have lower off-target potential than the use of two gRNAs (Figure 1A). By online efficiency prediction (Hsu et al., 2013; Mali et al., 2013), we identified five gRNA targets with fewer off-target effects on the host genome (Table S2) and constructed the corresponding CRISPR-Cas9 systems.

### Specific inactivation of HBV replication by CRISPR-Cas9

**Figure 2:**
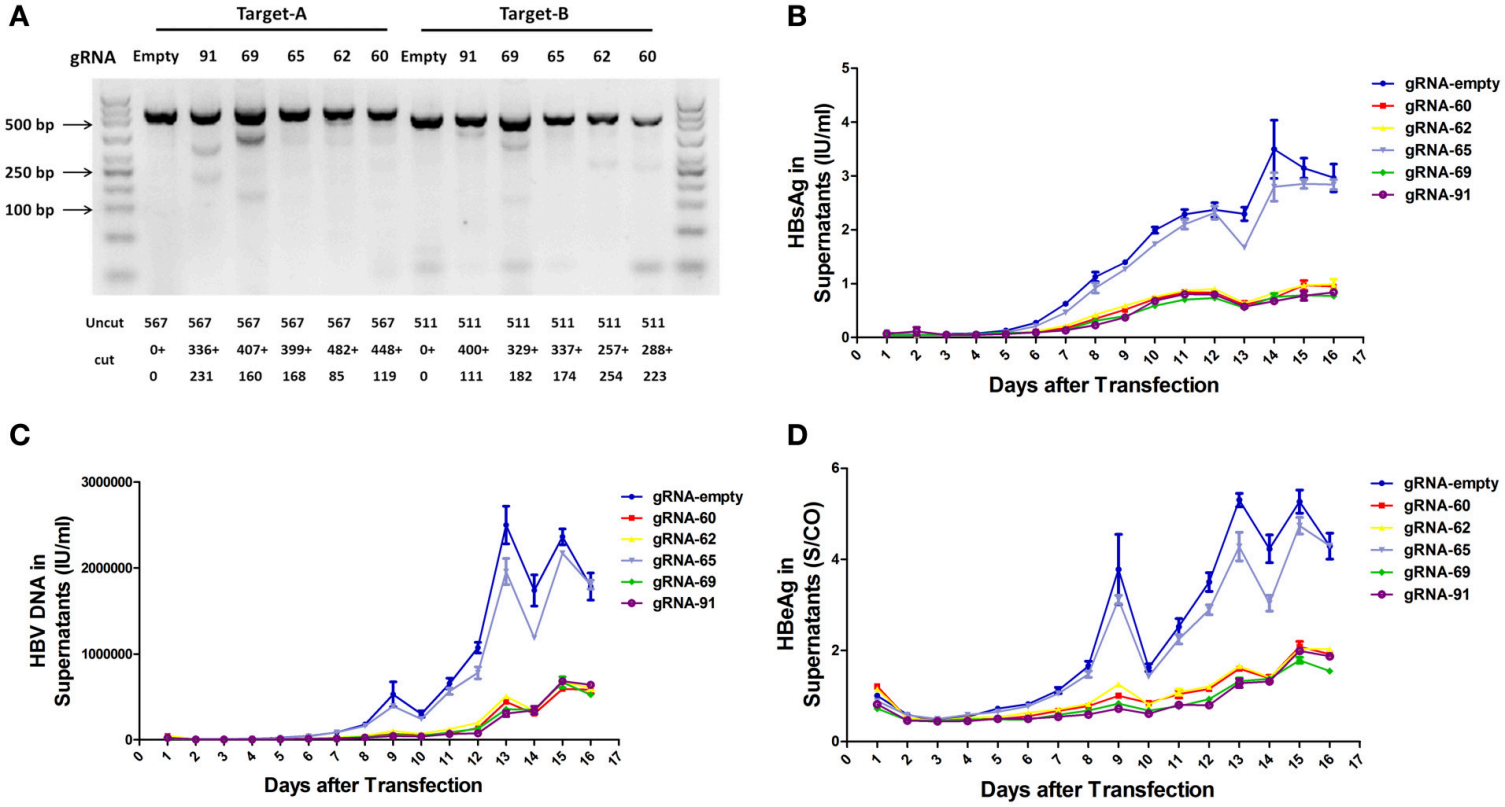
**Inhibition of both HBV antigen expression and HBV replication by CRISPR-Cas9 in A64 cells after transfection. (A)** DNA extracted from A64 cells transfected with gRNA-91, gRNA-69, gRNA-65, gRNA-62, and gRNA-60 was analyzed by a T7EI assay. Predicted sizes of uncut and cut bands are indicated. **(B–D)** Inhibition of HBsAg, HBV DNA, and HBeAg in cell culture supernatants at the indicated time points after transfection of A64 cells with gRNA-91, gRNA-69, gRNA-65, gRNA-62, and gRNA-60.

To assess the capacity to generate apparent cleavage at both ends of the integrated HBV DNA, we performed a mismatch-sensitive T7 endonuclease I (T7EI) assay (Ran et al., 2013) after transfecting the five gRNAs into the A64 cell line (Figure 2A). All of the systems introduced double-strand breaks (DSBs) into both ends of the integrated HBV DNA, but gRNA-69 was the most efficient system (Figure 2A). Next, to determine the knockout efficiency of the gRNAs in A64 cells, we used an ARCHITECT HBsAg and HBeAg reagent kit to determine the amounts of HBeAg and HBsAg in the cell culture supernatants on 16 consecutive days post-transfection. At day 9 after transfection, compared with that in the gRNA-empty group, HBeAg concentrations were reduced 83.13 ± 0.14% in the gRNA-91-treated group, 80.53 ± 2.43% in the gRNA-69-treated group, 70.71 ± 2.09% in the gRNA-62-treated group, and 76.50 ± 0.27% in the gRNA-60-treated group. HBsAg concentrations were reduced 87.38 ± 1.56% in the gRNA-91-treated group, 86.49 ± 1.79% in the gRNA-69-treated group, 80.07 ± 1.01% in the gRNA-62-treated group, and 82.55 ± 0.78% in the gRNA-60-treated group. HBV DNA concentrations were reduced 91.72 ± 1.55% in the gRNA-91-treated group, 89.21 ± 2.72% in the gRNA-69-treated group, 80.30 ± 1.30% in the gRNA-62-treated group, and 86.95 ± 1.93% in the gRNA-60 treated-group. It is noteworthy that the suppression of HBsAg and HBV DNA was slightly higher than that of HBeAg. During 16 consecutive days after transfection, HBV DNA, HBeAg, and HBsAg concentrations in culture supernatants were low in the gRNA-91, gRNA-69, gRNA-62, and gRNA-60 groups (Figures 2B,D) compared with those in the gRNA-empty group (Figure 2C). Neither the HBV DNA nor HBV antigens have been reduced in the cells transfected with gRNA-65. Moreover, at 10 and 14 day post-transfection, the entire group showed a drop-off in suppression of HBeAg and HBV DNA. This phenomenon was expected due to the loss cells during medium changing.

### CRISPR-Cas9 excised the full-length integrated HBV genome and disrupted HBV cccDNA

**Figure 3:**
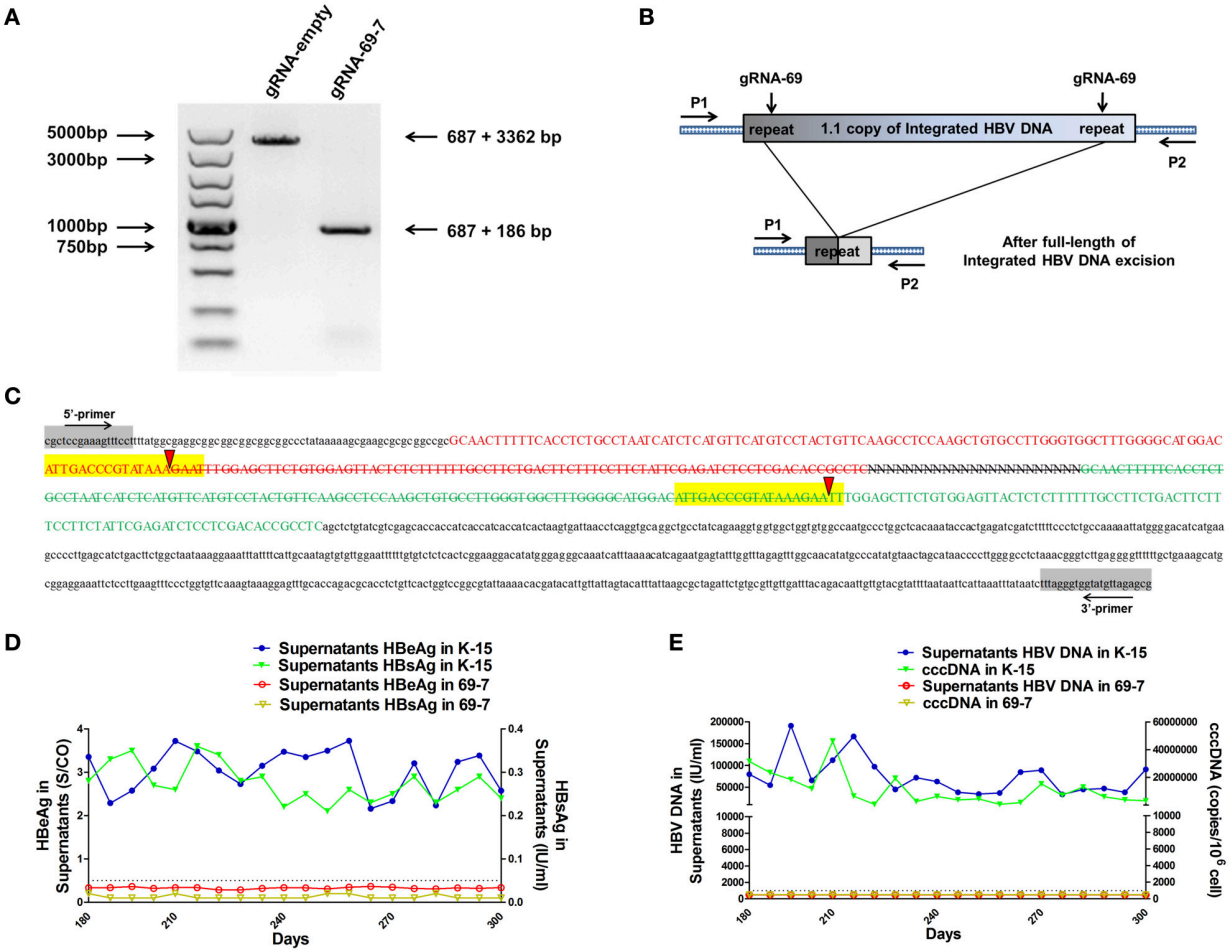
**CRISPR-Cas9/gRNA-69 efficiently removed the integrated HBV genome from a stable HBV cell line. (A)** Analysis of PCR amplicon lengths using a primer pair (P1 and P2) targeting the integrated HBV-flanking sequence revealed elimination of the full-length integrated HBV genome (3,173-bp), leaving one fragment (873-bp predicted segment from its flanking region). **(B)** Diagram showing excision of the full-length integrated HBV genome. The remaining fragment included the expected 687-bp from the integrated HBV flanking sequence and a 186-bp HBV repeat core region sequence. **(C)** Sanger sequencing of the remaining fragment (873-bp) showing the HBV flanking sequence (small letters, 687-bp) and the partial sequences (189 − 3 = 186-bp) of the integrated HBV repeat region B (green) and repeat region A (red) with a 3-bp deletion around the gRNA-69 targeting site (yellow-highlighted). Elimination of the full-length integrated HBV genome is indicated by a strikethrough. **(D,E)** The amounts of HBeAg, HBsAg and HBV DNA in cell culture supernatants and HBV cccDNA in the gRNA-empty-treated group (K-15) and gRNA-69-treated group (69-7) over 300 consecutive days. The HBsAg and HBeAg test results in the gRNA-69-treated group (69-7) were always under the negative threshold (0.05 IU/ml for HBsAg and 1 S/CO for HBeAg), and the amounts of HBV DNA and HBV cccDNA in the supernatants were always undetectable (<500 IU/ml for qPCR) in the gRNA-69-treated group (69-7). All viral markers in the gRNA-empty-treated group (K-15) remained at high levels.

We expected that expression of the gRNAs in A64 cells would result in the deletion of the entire 3,173-bp HBV genome between the A and B target sites. By long-range PCR analysis, we found one subclone, HepG2.A64-69-7 (A64 transfected with gRNA-69, hereafter referred to as “69-7”), which contained a complete deletion of the entire 3,173-bp integrated HBV genome (Figure 3A). An 873-bp fragment representing the predicted segment resulting from its flanking region was amplified (Figure 3A), suggesting gRNA-69 enabled Cas9 to excise the full-length integrated HBV genome segment. Sequence analysis demonstrated that the 873-bp fragment included 687-bp from the integrated pTriexHBV1.1-derived flanking sequence and a 186-bp HBV repeat core region sequence, with a 3-bp deletion at the gRNA-69 target site, which was expected to result from gRNA-69-guided cleavage and repair (Figure 3B).

Next, we performed quantitative analysis of HBsAg and HBeAg in the supernatant (Figures S1A,B) and real-time PCR analysis of supernatant HBV DNA and HBV cccDNA (Figures S1C,D). The HBV DNA in the supernatant from the gRNA-empty-treated group (subclone K-15) had a concentration of 775,033 ± 29,868 IU/ml at day 10 after continuous cultivation, whereas the concentration of the HBV DNA in the supernatant from the gRNA-69-treated group (subclone 69-7) was 420 ± 278 IU/ml. HBV cccDNA in subclone 69-7 was undetectable by qPCR (<500 copies/10^6^ cells), whereas HBV cccDNA in subclone K-15 had a concentration of 11,807,834 ± 3,431,702 copies/10^6^ cells (Figures S1C,D). Additionally, the amounts of HBsAg and HBeAg in the supernatant from subclone 69-7 were always below the negative threshold (0.05 IU/ml for HBsAg and 1 S/CO for HBeAg) for 10 consecutive days, whereas the amounts of HBsAg and HBeAg in the supernatant from subclone K-15 increased rapidly (Figures S1A,B). Next, to test whether this HBV clearance could be repaired, we conducted long-term observations of subclones 69-7 and K-15. In contrast to the high levels of HBeAg and HBsAg in the supernatant from subclone K-15, the amounts of HBeAg, HBsAg, and HBV DNA in the supernatant from subclone 69-7 remained below the negative critical values for 300 consecutive days; additionally, HBV cccDNA was undetectable in subclone 69-7. In contrast, all of these viral markers remained high in subclone K-15 for 300 consecutive days (Figures 3D,E).

### Whole genome sequencing of HBV-excised cell line

To assess possible off-target effects of CRISPR-Cas9 and the integrated subgenomic HBV DNA fragments in the original HBV-containing cell line (A64) and HBV-excised cell line (69-7), whole-genome sequencing (WGS) was performed. The average coverage was 71 folds in A64 and 67 folds in 69-7. In total, with human (GRCh37/hg19) genomes as reference sequences, we identified 4,981,628 SNPs, 914,579 indels and 24,344 SVs in A64 cells and 3,834,414 SNPs, 771,687 indels, and 16,719 SVs in 69-7 cells using GATK, v.2.8.1. Next, we performed an analysis of ±500-bp flanking each indel against predicted gRNA-69 off-target sites (Table S3). In total, we identified 6 indels in 50 potential off-target regions (Table 1), while four indels were identified in two cell lines and only two indels were different after gRNA-69 transfection, which suggests that gRNA-69 did not have significant off-target indels. Moreover, after comparing the SNPs and indels between HepG2.A64.69-7 and HepG2.A64 cells, we found that 1,175,423 SNPs and 154,931 indels were only present in the stable HBV cell line and not the HBV-excised cell line; 28,209 SNPs and 12,039 indels were present only in the HBV-excised cell line but not in the stable HBV cell line; 3,806,205 SNPs and 759,648 indels occurred in both samples. Thus, it means that the HBV-excised HepG2.A64.69-7 cell line missed millions of mutations compared with the stable HBV cell line HepG2.A64.

**Table 1:** **Detailed information on six called indels near gRNA-69 predicted off-target sites in A64 and 69-7 cells**.

**Name**	**gRNA-target**			**Indels**					**Genotype**	
	**Chromosome**	**Position**	**Strand**	**Chromosome**	**Position**	**Reference**	**Quality**	**Mutation**	**gRNA-empty**	**gRNA-69**
gRNA-69	18	76,276,328	+	18	76,276,322	T	4109.16	TC	1/1	1/1
gRNA-69	9	114,763,107	−	9	114,763,025	C	785.4	CTGA	0/1	0/1
gRNA-69	13	68,377,461	+	13	68,377,761	A	655.4	AT	0/1	0/1
gRNA-69	6	152,925,354	−	6	152,925,295	A	543.19	ATG	1/1	0/0
gRNA-69	15	96,449,419	+	15	96449337	A	3684.15	AAAAAG	0/1	1/1
gRNA-69	2	68,269,754	+	2	68,269,960	CGGGGGGGG	2285.21	C	1/1	1/1

**Figure 4:**
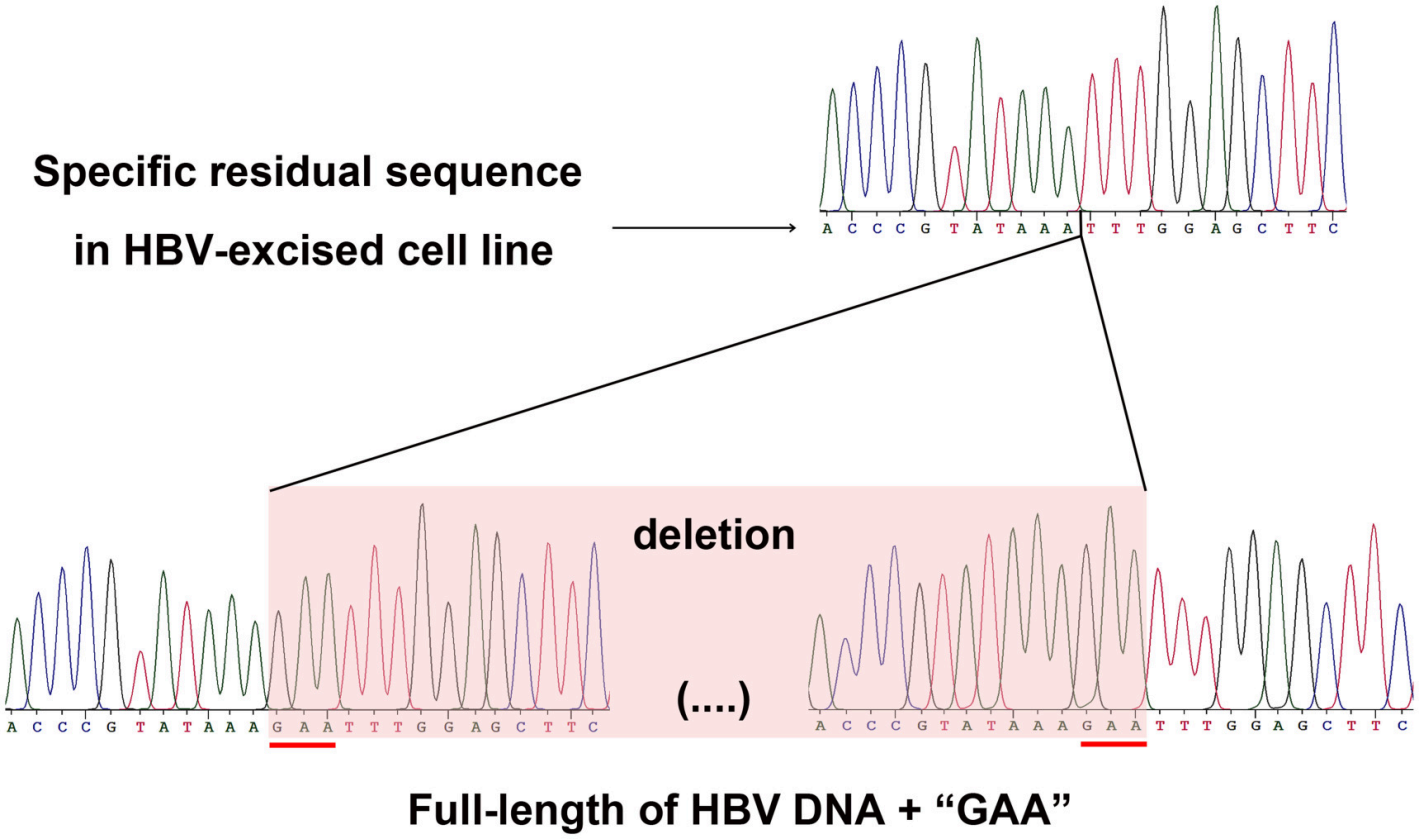
**Sanger sequencing of the gRNA-69/Cas9 target region both in the HBV-excised cell line 69-7 (upper half) and two ends of the full-length integrated HBV DNA in the stable HBV cell line A64 (lower half)**. The specific residual sequence with a three-nucleotide “GAA” deletion at the gRNA-69/Cas9 cleavage region in HBV-excised cell line 69-7 was used to distinguish it from the stable HBV cell line. The extra “GAA” deletion was marked by a red line in the stable HBV cell line A64.

As a proof that the HBV-excised monoclone 69-7 did not exist in a very small amount as contaminants in the stable HBV cell line A64 before gRNA-69 treatment, we found a specific residual sequence of gRNA-69/Cas9 cleavage (Figure 4), which confirmed that this monoclone was generated by gRNA-69/Cas9. Sanger sequencing of PCR products containing the excision site (Figure 3C) showed that after full-length HBV DNA excision by gRNA-69/Cas9 and non-homologous end joining (NHEJ) repair, three extra nucleotides “GAA” were deleted at the gRNA-69 cleavage site located in the HBV C region. Thus, we searched two 20-bp sequences (CCCGTATAAATTTGGAGCTT and CCGTATAAA**GAA**TTTGGAGC) that could distinguish the original and excised DNA in the raw sequencing data (coverage 60×) of the two samples. The results showed that before gRNA-69/Cas9 transfection, we could not find the residual sequence with the “GAA” deletion in the stable HBV cell line A64, while 11 reads containing the residual sequence with the “GAA” deletion were found in the 69-7 sequencing data. This residual sequence with the “GAA” deletion at the gRNA-69 targeting region not only indicated that the HBV-excised cell line was established by gRNA-69/Cas9, it also verified that this monoclone was not a contaminated clone in the A64 cell line.

## Discussion

CRISPR-Cas9 represents a potential means to radically cure chronic viral infections because this system was demonstrated to disrupt HBV cccDNA with favorable effects (Seeger and Sohn, 2014; Kennedy et al., 2015; Zhen et al., 2015a). Seeger et al. demonstrated that the use of CRISPR/Cas9 is currently the best method of functionally inactivating HBV cccDNA (Seeger and Sohn, 2016). HBV infection can induce HBV DNA fragment integration into the host genome, which had been observed in ~80% of cases of HBV-induced hepatocarcinogenesis (Bréchot, 2004; Hai et al., 2014). Integrated HBV DNA could lead to aberrant regulation of host gene expression (Jiang S. et al., 2012; Jiang Z. et al., 2012; Sung et al., 2012; Xu et al., 2014) and higher genomic instability (Cha and Dematteo, 2005). Many HBV integration events occurred near or within fragile sites and other repetitive regions, such as *TERT, FN1, MLL4, ROCK1, CCNE1, SENP5* (Jiang Z. et al., 2012; Sung et al., 2012; Hai et al., 2014), *Alu* sequences, and microsatellites, which are prone to tumor development and progression (Feitelson and Lee, 2007). Gounder et al. demonstrated that the reduced risk of HCC was not associated with HBsAg seroclearance, and speculated that it was probably associated with the existence of HBV cccDNA and integrated HBV DNA (Gounder et al., 2016). Here, as proof of concept, we employed the CRISPR-Cas9 system to eradicate HBV infection and eliminated the persistent HBV genome, including the full-length integrated HBV DNA and HBV cccDNA, in a stable HBV cell line. These findings indicated that CRISPR-Cas9 could not only provide a powerful path toward a radical or “sterile” HBV cure, but also provide a means of blocking carcinogenesis by eliminating HBV cccDNA and integrated HBV DNA.

Using gRNAs targeting the HBV core region, the level of HBsAg and HBV DNA was reduced by an equal level as that of HBeAg (Figures 2B–D). It had been considered as a global reduction in cccDNA in this case (Seeger and Sohn, 2014; Kennedy et al., 2015; Zhen et al., 2015b) for the HBV cccDNA rapid cleavage efficiency, resulting in a high percentage of linear DNAs that is not repaired but rather destroyed (Dong et al., 2015). However, the inhibitory effect on gRNA-directly targeted genes was stronger than the effect on other HBV antigens in previous studies (Lin et al., 2014; Kennedy et al., 2015). Since the functional full-length integrated HBV DNA is also a replication root in an HBV-transgenic cell model (Sells et al., 1987) and these four effective gRNAs targeted both ends of the full-length integrated HBV DNA, we reasoned that this more thorough clearance of HBV expression was not only caused by the global reduction of cccDNA, but also by the removal of the full-length integrated HBV DNA. Another possible reason for this overall reduction was that the region we targeted, which contained the viral enhancer, X gene, and core gene(Sung et al., 2012), is essential for HBV replication or even integration (Zoulim and Locarnini, 2009). Since the specific mechanisms of HBV replication and integration remain controversial, we expect that this region will be an area of focus for future research.

In a monoclone of the stable HBV cell line 69-7, the full-length integrated HBV genome was excised. It is the first time, to our knowledge, that the HBV infection in a stable HBV cell line was removed thoroughly and completely using CRISPR-Cas9. At first, we aimed to value the off-target effect of gRNA-69. Unexpectedly, after sequencing the whole genome, we identified 4,981,628 SNPs in HepG2.A64 cells and only 3,834,414 SNPs were detected in the HBV-excised 69-7 cell line. We found that 1,175,423 SNPs in HepG2.A64 were absent after HBV excision. There may be two possibilities to explain this finding. Firstly, those abundant missing SNPs may be due to limited WGS coverage. Secondly, as many studies have demonstrated that HBV proteins such as HBx, HBsAg and core proteins could interfere with DNA repair [i.e., the NER (Jaitovich-Groisman et al., 2001; Lieber, 2010), BER (van de Klundert et al., 2012) or ATR (Rakotomalala et al., 2008; Wang et al., 2008)] pathways, this could have exacerbated DNA damage, by interfering with checkpoint activation, promoting cell cycle progression and ultimately leading to genetic aberrations. We speculate that those missing SNPs could have been eliminated by the re-activated DNA repair mechanism of the host. However, the specific mechanisms of HBV integration and HBV pathogenesis remain controversial, and this suggestion still needs further research.

In addition, as we established only one HBV-excised subclone without any HBV replication markers and missing abundant SNPs, there is a possibility that this HBV-excised subclone we selected is just the HepG2 cell line contaminated in HepG2.A64. To rule out this possibility, we used the WGS results with a specific residual sequence of the gRNA-69/Cas9 cleavage to evaluate the contaminant. The results showed that the HBV-excised monoclone 69-7 did not exist as contaminants in the stable HBV cell line A64 before transfection and it was generated by gRNA-69/Cas9. Unlike previous studies using CRISPR-Cas9 to disrupt HBV, we not only disrupted HBV cccDNA but also swept the full-length of integrated HBV DNA in the stable HBV cell line. In the HBV-excised cell line 69-7, HBV cccDNA, supernatant HBV DNA, HBsAg, or HBsAg could not be detected for 10 consecutive months. This showed that once the root of HBV replication in a stable HBV cell line had been eradicated, and HBV infection was persistently undetectable without any treatment. As the development of the next generation of sequencing technology, we could specific locate the integrated HBV DNA fragments. The developed online gRNA predicting tool could be used to design gRNAs with less off-target effect. Besides, in recent years, Adeno-Associated Virus Vectors (AAV) has captured much attention as a gene delivery system for treating human disease caused by a gene loss or mutation. The advantages of the AAV delivery scheme include its low toxicity and sustained gene expression, which can extend to 12 months after a single administration. Those properties mean that completely eradication of HBV infection in clinical CHB patients will be achieved in the near future.

## Author contributions

HL, CS, YS, HS, and SQ conceived the study. HL, SW, and CS cultured cells and isolated genomic DNA. CS, HL, YH, HBL, YT, and LY processed experiments and analyzed the WGS data. QL, PL, YL, ML, JZ, CY, XY, LJ, JX, LW, RH, and DX shared regents and materials. All authors read and approved the final manuscript.

## Funding

This work was supported by grants from the National Nature Science Foundation of China (81371854), the Beijing Nature Science Foundation (7162145), and the Innovation Foundation of AMMS (2015CXJJ26).

### Conflict of interest statement

The authors declare that the research was conducted in the absence of any commercial or financial relationships that could be construed as a potential conflict of interest.

## Abbreviations

HBV: hepatitis B virus
NAs: nucleoside/nucleotide analogs
cccDNA: covalently closed circular DNA
CRISPR: clustered regularly interspaced short palindromic repeat
gRNA: guide RNA
PAM: protospacer adjacent motif
DSBs: double-strand breaks
HBS: hepatitis B surface antigen
indel: insertion or deletion
SD: standard deviation
NHEJ: Non-homologous end joining
PSAD: plasmid safe ATP-dependent DNase
RCA: rolling circle amplification.
