# Supplementary material for: Removal of Integrated Hepatitis B Virus DNA Using CRISPR-Cas9
Source: Front Cell Infect Microbiol. 2017 Mar 22;7:91. doi: 10.3389/fcimb.2017.00091 (PMC5360708; doi:10.3389/fcimb.2017.00091)
Supplement: Supplementary file 1 [file Table1.docx]

**Supplementary Materials**

Table S1. Primers used in this study.

Table S2. Sequences of gRNAs used in this study.

Table S3. Top 50 potential off-target sites for gRNA-69 in the human genome.

Figure S1. Levels of HBV antigen expression and HBV replication in HBV excised cell line.

Table S1. Primers used in this study

| **Primers** | **Sequence (5'-3')** |
| --- | --- |
| **P1** | CGCTCCGAAAGTTTCCTT |
| **P2** | CGCTCTAACATACCACCCTAAA |
| **P3** | GAGTGCGAATCCACACTCC |
| **HBSF** | TCACAATACCGCAGAGTC |
| **HBSR** | ACATCCAGCGATAACCAG |
| **A1ATF** | TTCCCTGGTCTGAATGTGTG |
| **A1ATR** | ACTGTCCCAGGTCAGTGGTG |
| **Target A region F** | TCCGAAAGTTTCCTTTTATGGCG |
| **Target A region R** | GGGGCATTTGGTGGTCTGTA |
| **Target B region F** | TGTGCACTTCGCTTCACCTC |
| **Target B region R** | ACCACCTTCTGATAGGCAGC |

Table S2. Sequences of CRISPR gRNAs used in this study

| Name | gRNA sequence (5’-3’) | PAM |
| --- | --- | --- |
| gRNA-91 | TCGAGGAGATCTCGAATAGA | AGG |
| gRNA-69 | ATTGACCCGTATAAAGAATT | TGG |
| gRNA-65 | AAGCTCCAAATTCTTTATAC | GGG |
| gRNA-62 | AACATGAGATGATTAGGCAG | AGG |
| gRNA-60 | AAGCCTCCAAGCTGTGCCTT | GGG |

Table S3. Top 50 potential off-target sites for gRNA-69 in the human genome.

| sequence | score | UCSC gene | locus（hg19） |
| --- | --- | --- | --- |
| ATTAACCAGGATAAAGAATTCAG | 2.3 |  | chr4:-99860095 |
| ATTGACACGTATAAAGAAATCAG | 2.2 |  | chr18:-35216827 |
| AGTGACTTGTATAAAGAATTCAG | 1.7 |  | chr6:-20203147 |
| ATTGTCCAATATAAAGAATTTAG | 1.5 |  | chr5:-102548578 |
| ATTCACCAGTGTAAAGAATTTAG | 1.4 |  | chr16:+22430585 |
| TCTGTCCTGTATAAAGAATTTAG | 1.4 |  | chr18:+76276328 |
| ATTTACTCTTATAAAGAATTAAG | 1 |  | chr3:-182619149 |
| ATTTACACCTATAAAGAATTCAG | 1 |  | chr9:+109244158 |
| ACACACACGTATAAAGAATTTGG | 0.9 |  | chr18:+37488373 |
| ATAGCCCTTTATAAAGAATTTGG | 0.8 |  | chr4:-126738295 |
| CTAGAGCCGCATAAAGAATTAGG | 0.8 |  | chr1:-162089465 |
| CTTGGCCAGTATAAAGAATGAAG | 0.7 |  | chr15:+101305146 |
| AATTACCAGTATAAAGAATGAGG | 0.7 |  | chr18:+23440812 |
| GTTAACCCGAATAAAGAATGAAG | 0.7 |  | chr9:+121424866 |
| TTTGCCGCATATAAAGAATTCGG | 0.6 |  | chr15:-51407115 |
| CTTGAAGGGTATAAAGAATTTAG | 0.6 | NM_001040453 | chr15:+59149441 |
| AGAGACCTGTATGAAGAATTCAG | 0.6 |  | chr20:-55299867 |
| ATTTACTGTTATAAAGAATTTGG | 0.6 |  | chr1:+242662660 |
| TTTGACCAGTATAAATAATTTGG | 0.6 |  | chr9:-24544016 |
| ATTAACCAGCATAAAGCATTGAG | 0.5 |  | chr12:-73601554 |
| ATAGCCCAGTATAAAGAAATCAG | 0.5 | NM_014877 | chr17:-65174773 |
| ATCTAACCTTATAAAGAATTTGG | 0.5 |  | chr10:+84032260 |
| ACTGACCAGAATAAAGAAATCAG | 0.5 |  | chr20:+11238920 |
| ATTGGCCTTTTTAAAGAATTAAG | 0.5 |  | chr9:+26839165 |
| ATTAACCAGAATAAAGAAATCAG | 0.5 |  | chr9:-114763107 |
| CTTGAGCCGGTTAAAGAATTTGG | 0.4 | NM_032175 | chr5:-72861839 |
| GTTGACCAATATAAAGAATAAAG | 0.4 |  | chr4:+24383299 |
| ATTCAGCCGACTAAAGAATTCAG | 0.4 |  | chr13:+68377461 |
| ATAGAACGGTACAAAGAATTCAG | 0.4 |  | chr8:-93435112 |
| ATTATCCCTTATAAAGAATGTGG | 0.4 |  | chr1:+23635893 |
| CTTGACCCTGAAAAAGAATTAAG | 0.4 |  | chr1:-50586221 |
| ATAGAACAGTATAAAGAATAAAG | 0.4 |  | chr6:-152925354 |
| ATTTAACTGTATAAAGAATGCAG | 0.4 |  | chr15:+100232025 |
| ATAGACATGTATAAAGTATTCAG | 0.4 |  | chr12:-29502612 |
| ACTGACCCTAAAAAAGAATTCAG | 0.4 |  | chr10:+123571197 |
| ATTTACGTGTATAAAGTATTTAG | 0.4 |  | chr11:+28138502 |
| ATTCACCCCAATAAAGAATACAG | 0.4 |  | chr15:+96449419 |
| AGTGACCTGGATAAGGAATTTGG | 0.4 |  | chr13:-99491174 |
| ATCGACCAGTTTAAAGAATCAAG | 0.4 |  | chr8:+27734865 |
| ATTGAACAGAATAAAGAATAAAG | 0.4 |  | chr12:-80048739 |
| TTTGCCACGTATAAAGAACTTGG | 0.4 |  | chr13:+110932471 |
| ACTAACCCCTATGAAGAATTAGG | 0.3 |  | chr7:-144945562 |
| AATGATCAGTATGAAGAATTCAG | 0.3 |  | chr7:+110637886 |
| ATGGATGCATATAAAGAATTAAG | 0.3 |  | chr10:+62666057 |
| ATTGATTTATATAAAGAATTAAG | 0.3 |  | chr1:-232628257 |
| ACTGACCTATATAAAGAAATAAG | 0.3 | NM_001190263 | chr2:+68269754 |
| ACTGACCTATATAAAGAAATAAG | 0.3 |  | chr10:+81800110 |
| ACTGACCTATATAAAGAAATAAG | 0.3 |  | chr10:+32799974 |
| ACTGATCAGTATAAAGAACTAGG | 0.3 |  | chr2:+125039888 |
| ATTGAATGGTCTAAAGAATTCAG | 0.3 |  | chr13:-73013453 |


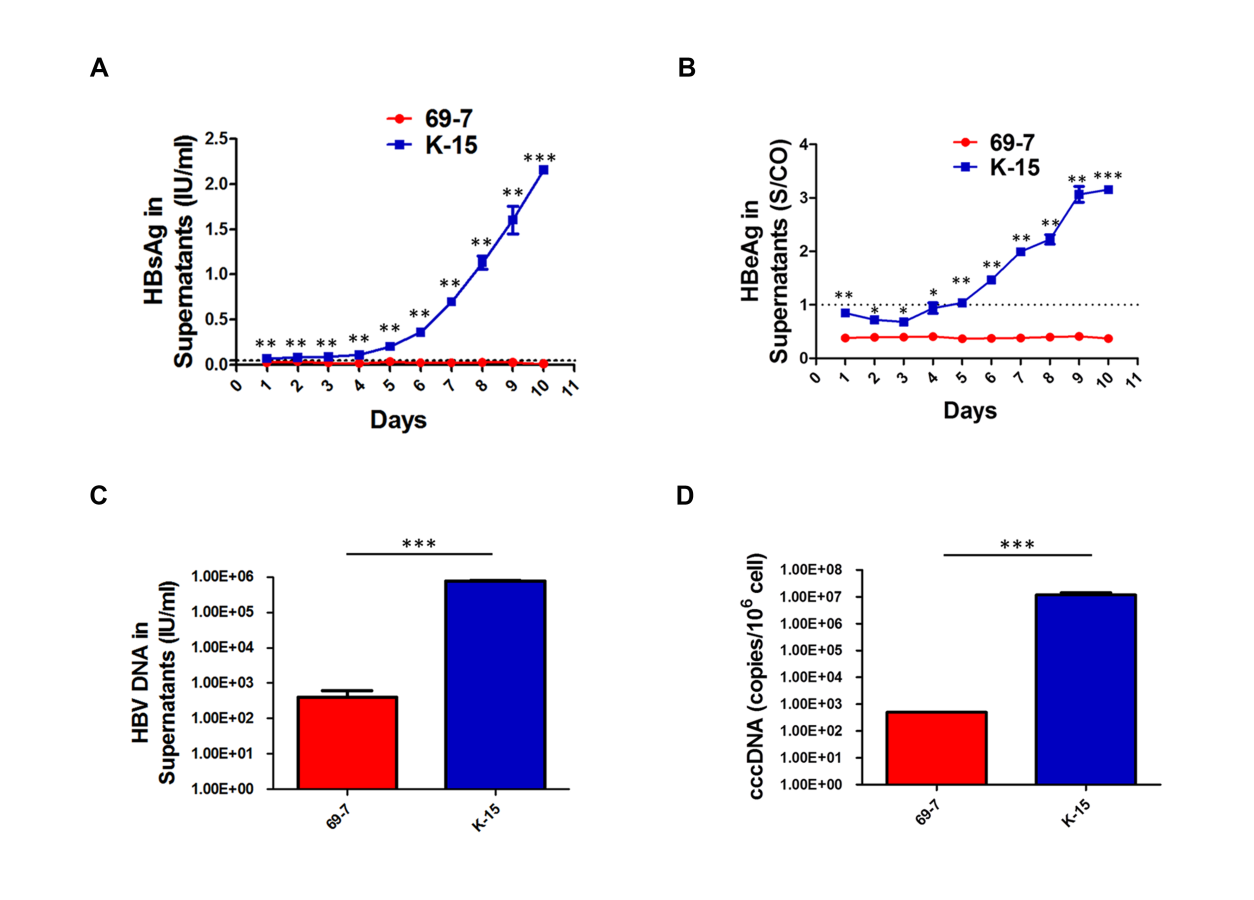


**Figure S1.** Levels of HBV antigen expression and HBV replication in HBV excised cell line. **(A, B)**Titers of HBsAg and HBeAg in cell culture supernatants during 10 consecutive days following establishment of HBV excised cell line 69-7. The HBsAg and HBeAg test results were always negative (HBsAg <0.05 IU/ml, HBeAg <1 S/CO) while the amounts of supernatant HBsAg and HBeAg in K-15 increased rapidly. **(C)** The amount of HBV DNA in 69-7 was 2,000-fold lower than that in K-15. **(D)** The amount of cccDNA in 69-7 were undetectable (<500 IU/ml for qPCR). Data represent means ± SD from three independent experiments performed in triplicate. Statistically significant differences are indicated by asterisks (*P < 0.05, **P < 0.01, ***P < 0.001).
